# A flexible routing scheme for patients with topographical disorientation

**DOI:** 10.1186/1743-0003-4-44

**Published:** 2007-11-28

**Authors:** Jorge Torres-Solis, Tom Chau

**Affiliations:** 1 Institute of Biomaterials and Biomedical Engineering, University of Toronto, Toronto, ON, Canada; 2 Edward S. Rogers Sr. Department of Electrical & Computer Engineering, University of Toronto, Toronto, ON, Canada; 3 Bloorview Research Institute, Toronto, ON, Canada

## Abstract

**Background:**

Individuals with topographical disorientation have difficulty navigating through indoor environments. Recent literature has suggested that ambient intelligence technologies may provide patients with navigational assistance through auditory or graphical instructions delivered via embedded devices.

**Method:**

We describe an automatic routing engine for such an ambient intelligence system. The method routes patients with topographical disorientation through indoor environments by repeatedly computing the route of minimal cost from the current location of the patient to a specified destination. The cost of a given path not only reflects the physical distance between end points, but also incorporates individual patient abilities, the presence of mobility-impeding physical barriers within a building and the dynamic nature of the indoor environment. We demonstrate the method by routing simulated patients with either topographical disorientation or physical disabilities. Additionally, we exemplify the ability to route a patient from source to destination while taking into account changes to the building interior.

**Results:**

When compared to a random walk, the proposed routing scheme offers potential cost-savings even when the patient follows only a subset of instructions.

**Conclusion:**

The routing method presented reduces the navigational effort for patients with topographical disorientation in indoor environments, accounting for physical abilities of the patient, environmental barriers and dynamic building changes. The routing algorithm and database proposed could be integrated into wearable and mobile platforms within the context of an ambient intelligence solution.

## Background

Topographical orientation is the ability to orient oneself within the environment and to navigate through it to specific destinations [1]. Through recent magnetic resonance imaging studies, specific structures such as the parahippocampal gyrus [2], parietal cortex [3] and temporal cortical areas [4] have been implicated as neural mechanisms for topographical orientation. It is generally agreed that in normative way-finding, humans employ a number of different way-finding strategies, including landmark recognition, route learning and map-like representations [5]. The particular choice of strategy is dependent on the individual's developmental age, the familiarity with the environment, the manner by which the environment was introduced, the level of detail in the environment and the specific navigational task at hand.

Topographical disorientation generally refers to the family of deficits in orientation and navigation in the real environment. Aguirre and D'Esposito [5] note that difficulties in way-finding may arise from a variety of different lesions or injuries and provide a well-accepted taxonomy of this disorder. For example, people living with post-traumatic effects of brain injury often have symptoms such as weakness in visual scanning skills, complex attention, prospective memory and sequential processing [6]. These symptoms can lead to problems of interaction with and perception of the surrounding environment, even several years after the injury [7,8]. It is well recognized that topographical disorientation [9] and spatial navigation deficits [10] are common sequelae of brain injury.

Current therapies for topographical disorientation, such as simple mnemonic techniques [11] or compensatory wayfinding strategies [12], often require the presence of an occupational therapist over extended periods of time. Consequently, conventional therapies are both time and human resource intensive. Recent developments in ambient intelligence suggest that navigational support to patients with topographical disorientation among other disabilities, may be provided by smart technologies embedded in the environment and wearable devices [13-15]. An ambient intelligence (AmI) system would be aware of the patient's location and physical abilities as well as the building's structural layout. The AmI system would provide context-specific navigational assistance in the form of visual or verbal cues through an augmented reality interface. As a first step towards such a system, Chau et al. [16] reported a desktop augmented reality system for patients with acquired brain injury where pictures of navigation decision points inside a building were superimposed on the real environment to encourage the retraining of wayfinding skills. In terms of an embedded implementation, Blache et al. [17] recently proposed a full-fledged ambient intelligence navigation solution using a relational database to maintain information about the building structure and mobile entities within, a Dijkstra routing engine for navigation, WLAN for user localization and a PDA as the user interface.

In this paper, we focus specifically on the central processing module of an indoor ambient intelligence system, namely the routing engine. In particular, we address some previously unconsidered challenges of routing patients with topographical disorientation. Unlike data packets, each patient has unique abilities and limitations, implying that a given environment may present different challenges to different users. Furthermore, the indoor environment may be dynamic: certain pathways may become unavailable due to facility cleaning and maintenance, renovation projects, special events or emergency closures. This is especially true in busy hospital environments. Finally, due to spatial disorientation, patients may make errors along the recommended route. These challenges imply that a single set of navigational instructions would not suffice and some form of dynamic and patient-specific routing is required.

### Selective routing

In typical routing schemes such as the Routing Information Protocol (RIP) [18,19], the Open Shortest Path First (OSPF) protocol [20,21] or the Border Gateway Protocol version 4 (BGP-4) [22], all the routed elements (e.g., packets) are processed in a uniform manner. In the present case, however, we intend to route patients, each with unique characteristics. The minimum distance route assuming uniformly processed packets is therefore not necessarily the optimal solution.

In recent years, some authors have suggested selective routing for packet networks as a means to implement Quality of Service (QoS) mechanisms for different types of traffic on the Internet [23,24]. These routing schemes take into account the type of traffic that the packet carries based on a tag (packet context) and the topology of the network. In other words, each packet is given individualized treatment, either on the basis of its content or to maximize network efficiency. The selective routing idea is an appealing approach to route patients based on their individual characteristics and attributes of the environment. To the best of our knowledge, selective routing of human subjects has not been previously reported in the literature.

## Proposed method

The proposed patient routing scheme consists of a database, weighted connected graph and a routing algorithm. Each component will be described in turn.

### Database

Information pertaining to patient disability, the building layout and environmental barriers along paths of travel are organized into a relational database consisting of three tables.

An adjacency table captures the building layout which is represented by a connected graph. The content of this table is the upper triangle of an adjacency matrix for the graph, coded as triplets of the form (node A, node B, ID), indicating a connection (edge) between node A and node B. The ID element is a numerical identifier which serves as a key into an accompanying context table.

A context table embodies information about environmental barriers. This table holds the attributes of each edge or link of the graph in the form of numerical weights. Example attributes include the physical distance of the link, the tread length and rise height of each step and the number of steps in a staircase, the level of illumination (luminous intensity) or the multiplicity of nearby permanent landmarks. In practice, the values of these attributes could be physically measured or derived from architectural drawings.

A patient table captures vital information about the patient, namely, personal data such as name, age, sex, contact information, and most importantly, individual ability levels. The latter were captured via a set of weights denoting the individual's ability to negotiate stairs, ramps, elevators, poor illumination and other potential barriers to mobility. A patient is defined in the database by specifying the attribute values for the fields in the patient table. For example, a patient without disability might have zero values for stair difficulty, ramp difficulty and low illumination fields while a patient with impaired mobility might have a large positive value for the stair difficulty field. In practice, the weights in the patient table might be determined from standardized assessments for gross motor function (e.g., GMFCS [25]), visual acuity or dynamic balance (e.g., center-of-mass kinematics [26]).

**Figure 1:**
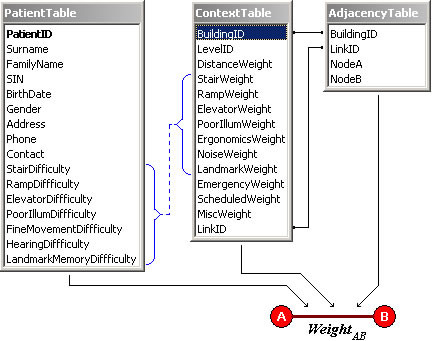
A sample database structure for the proposed method. Information from the patient and context tables are combined to generate a patient-specific weight for each link.

Figure 1 shows a graphical representation of a simple database with some sample fields. In our implementation, the database was implemented in MySQL.

### Generation of a weighted connected graph

As alluded to earlier, a connected graph represented the target building. Deriving the graph involved strategically placing nodes and estimating weights, each of which is explained below.

#### Placement of nodes

**Figure 2:**
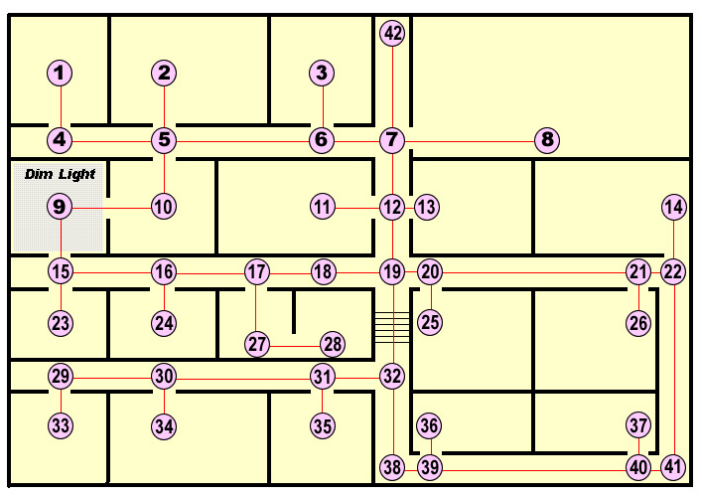
A connected graph generated from a building floor plan. This figure shows the synthetic building generated and its connected graph representation, which will be used throughout most of the subsequent experiments.

Mapping schemes designed to represent a building floor plan with a connected graph have been previously proposed [27-29]. Our mapping is an adaptation of the method of Belkhous *et al.*[28]. In the proposed scheme, a node is placed on the floor plan at each decision point, that is, any physical space where the patient is presented with a navigational choice. Figure 2 exemplifies a connected graph generated from one level of a building floor plan. Nodes have been placed at decision points, which may be doorways (e.g., node 17), corners (e.g., node 38), the interior of a room (e.g., node 1) or the intersections of hallways (e.g., node 7). Several decision points can be placed in large open areas if desired.

Nodes can also be placed on either side of potential physical barriers as exemplified by nodes 19 and 32, which encompass a staircase in Figure 2. Placing nodes in this way, facilitates the assignment of weights to the barriers. Nodes that represent physically connected spaces are joined with an edge. A numerical weight is then assigned to each link as described in the next section.

#### Estimation of edge weights

The weight on each link is a function of the weights for patient ability levels (patient table) and barriers in the building (context table). Generally, when a link contains an environmental barrier which unduly challenges a patient, the corresponding weight should be large. Clearly, the weight function is not unique. A simple linear function for determining the weight on a link A-B for a given patient is exemplified below.

WeightAB=DistanceWeight+∑i=1n(BuildingBarrieri×PatientDifficultyi)+(EmergencyFlag×EmergencyWeight)+(TimerFlag×ScheduledWeight)+(MiscFlag×MiscWeight)
 MathType@MTEF@5@5@+=feaafiart1ev1aaatCvAUfKttLearuWrP9MDH5MBPbIqV92AaeXatLxBI9gBaebbnrfifHhDYfgasaacPC6xNi=xI8qiVKYPFjYdHaVhbbf9v8qqaqFr0xc9vqFj0dXdbba91qpepeI8k8fiI+fsY=rqGqVepae9pg0db9vqaiVgFr0xfr=xfr=xc9adbaqaaeGacaGaaiaabeqaaeqabiWaaaGcbaqbaeaabmWaaaqaaiabdEfaxjabdwgaLjabdMgaPjabdEgaNjabdIgaOjabdsha0naaBaaaleaacqWGbbqqcqWGcbGqaeqaaaGcbaGaeyypa0dabaGaemiraqKaemyAaKMaem4CamNaemiDaqNaemyyaeMaemOBa4Maem4yamMaemyzauMaem4vaCLaemyzauMaemyAaKMaem4zaCMaemiAaGMaemiDaqNaey4kaSYaaabCaeaacqGGOaakcqWGcbGqcqWG1bqDcqWGPbqAcqWGSbaBcqWGKbazcqWGPbqAcqWGUbGBcqWGNbWzcqWGcbGqcqWGHbqycqWGYbGCcqWGYbGCcqWGPbqAcqWGLbqzcqWGYbGCdaWgaaWcbaGaemyAaKgabeaakiabgEna0kabdcfaqjabdggaHjabdsha0jabdMgaPjabdwgaLjabd6gaUjabdsha0jabdseaejabdMgaPjabdAgaMjabdAgaMjabdMgaPjabdogaJjabdwha1jabdYgaSjabdsha0jabdMha5naaBaaaleaacqWGPbqAaeqaaOGaeiykaKcaleaacqWGPbqAcqGH9aqpcqaIXaqmaeaacqWGUbGBa0GaeyyeIuoaaOqaaaqaaiabgUcaRaqaaiabcIcaOiabdweafjabd2gaTjabdwgaLjabdkhaYjabdEgaNjabdwgaLjabd6gaUjabdogaJjabdMha5jabdAeagjabdYgaSjabdggaHjabdEgaNjabgEna0kabdweafjabd2gaTjabdwgaLjabdkhaYjabdEgaNjabdwgaLjabd6gaUjabdogaJjabdMha5jabdEfaxjabdwgaLjabdMgaPjabdEgaNjabdIgaOjabdsha0jabcMcaPiabgUcaRiabcIcaOiabdsfaujabdMgaPjabd2gaTjabdwgaLjabdkhaYjabdAeagjabdYgaSjabdggaHjabdEgaNjabgEna0kabdofatjabdogaJjabdIgaOjabdwgaLjabdsgaKjabdwha1jabdYgaSjabdwgaLjabdsgaKjabdEfaxjabdwgaLjabdMgaPjabdEgaNjabdIgaOjabdsha0jabcMcaPaqaaaqaaiabgUcaRaqaaiabcIcaOiabd2eanjabdMgaPjabdohaZjabdogaJjabdAeagjabdYgaSjabdggaHjabdEgaNjabgEna0kabd2eanjabdMgaPjabdohaZjabdogaJjabdEfaxjabdwgaLjabdMgaPjabdEgaNjabdIgaOjabdsha0jabcMcaPaaaaaa@EE71@

The variables correspond to the fields in the patient and context tables portrayed in Figure 1. The *DistanceWeight *represents the physical distance between nodes *A *and *B*. The *BuildingBarrier*_*i *_variable corresponds to the stair, ramp, elevator or illumination weights while *PatientDifficulty*_*i *_denotes the corresponding patient ability level. For example, if the patient has impaired vision (high weight value for poor illumination difficulty in the patient table) and the link A-B denotes a dimly lit corridor (high weight value for poor illumination in the context table), then the product of the corresponding weights will make a large contribution to the overall weight on the link. *EmergencyFlag*, *TimerFlag *and *MiscFlag *are flags that indicate the occurrence of, respectively, an emergency code (e.g., fire), a time-dependent event (e.g., closure of a certain doorway at a specific time) and other miscellaneous situations which may affect patient routing. These flags might be toggled by alarm or monitoring systems within the building.

In this manner, the connected graph has different edge weights for different patients negotiating the same building. The final weighted graph is used in the determination of the optimal route. Evidently, links with large weights relative to those on other links are not favoured during routing.

### Routing scheme

Once we have a weighted graph representing the building layout, patient abilities and environmental barriers, a routing scheme can be deployed. Different optimization algorithms have been proposed for calculating the shortest path between two connected nodes in a graph. The Dijkstra algorithm, a time-honored graph-theoretic method [30] has been widely applied for routing packets in communication networks, and is still widely used by core routers, implemented in the 'Open Shortest Path First' (OSPF) routing algorithm [20,21]. It is also commonly applied for routing human subjects [17], mobile elements (i.e. robots) and virtual or simulated subjects in labyrinths and maps [27,31,28]. We therefore invoked the Dijkstra algorithm [30], which was programmed in PERL for simplicity of data management. Unlike conventional implementations that rely on a static graph, our approach uses a dynamically changing graph. Recall that there is a navigational choice at each node. Whenever the user reaches a new node, the routing algorithm references the context table in the database to obtain an up-to-date status of the indoor environment. With the current and destination nodes and most up-to-date estimation of edge weights as inputs, the algorithm returns the path of minimal cost. In this way, the "optimal" route in terms of minimal distance and best fit between environmental context and patient ability is found dynamically. Recalculating the route at every node has been previously proposed as a strategy to account for human mistakes [27]. However, previous work did not simultaneously accommodate environmental changes which may alter the building map and consequently, the graph structure.

## Simulations

### Patient simulator

We created a program to simulate a patient navigating through a building by following the directions given by the Dijkstra engine. To model patient disorientation, we defined a confusion probability, *P*_*C*_, that is, the probability of randomly selecting the next node rather than that recommended by the Dijkstra engine. The simulation program accepts as inputs the origin and destination nodes and the confusion probability. The patient simulation program with a confusion probability, *P*_*C*_, operated as follows.

1. The patient starts at a given source node.

2. The program generates a random number, *X*, between 0 and 1 from a uniform distribution.

(a) If *X *<*P*_*C*_, a random navigational decision is made. The program consults the interconnection map (adjacency table) to determine the number of possible paths emanating from the current node. One of the available adjacent nodes is randomly selected as the next position of the patient. Note that it is possible that either the path suggested by the Dijkstra algorithm or the path back to the patient's previous location might be selected.

(b) Otherwise, if *X *> *P*_*C*_, the navigational decision is per the Dijkstra recommendation. The

Dijkstra algorithm finds the optimal route using the current node as the origin node. The simulation program consults the context table to account for any recent changes in the environment. The patient will move to the next node as indicated by the Dijkstra engine.

3. Step 2 is repeated upon arrival at each new node until the patient reaches the destination node.

### Simulation of patients with topographical disorientation

**Figure 3:**
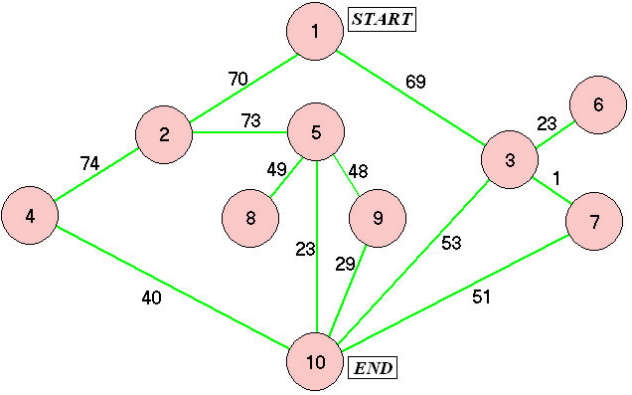
Connected graph used for routing simulated patients with topographical disorientation.

A connected graph with 10 nodes and different weights for the links was constructed as shown in Figure 3. We simulated patients with five different confusion levels, each traveling from node 1 to node 10. The minimum distance path was 1-3-7-10. The confusion probabilities were 0.25, 0.5, 0.75, 0.90 and 1.0.

The last patient (*P*_*C *_= 1.0) served as the benchmark subject who did not follow any navigational instructions and simply wandered randomly around the building until he stumbled upon the destination node. Wandering behaviour has been previously observed in patients with acquired brain injury [9]. Each patient was simulated 1000 times to account for route variations arising from random navigation when *P*_*C *_≠ 0. For the patient who followed every navigational instruction, i.e., *P*_*C *_= 0, the Dijkstra-suggested optimal path was unique and hence this patient was simulated only once. The number of nodes traversed between source and destination, the travel cost and the number of random decisions were recorded for each trial. After 1000 trials, the above data from patients with decreasing confusion probability, were compared against the random walk (*P*_*C *_= 1) results using a Wilcoxon rank sum test due to the non-gaussian data distributions.

### Simulation of patients with physical disabilities

This experiment intended to demonstrate how the proposed routing scheme accommodates patients with different abilities. We defined three different virtual patients, all with *P*_*C *_= 0. The first virtual patient had no impairments, i.e. null weights for all limitation attributes. The second virtual patient had a mobility limitation, with a high value for the "stair difficulty" field in the patient table. This patient might use a mobility aid and cannot easily negotiate stairways. The third virtual patient was characterized by a high value for the "poor illumination" field in the patient table, indicating the presence of a visual impairment. This latter patient should avoid rooms with inadequate illumination. Aside from the specified limitations, all other fields relating to patient disability were set to zero. Recall that the edge weights of the connected graph take into account patient and building attributes. Hence, each virtual patient was associated with a uniquely weighted graph representation of the building. This customization allows the routing algorithm to find the best route for a particular patient in a specific building, according to the patient's abilities and the current internal environmental conditions.

The virtual patients walked through the map depicted in Figure 2. In particular, we selected two paths, each of which traversed a space with a targeted physical barrier, i.e. a stairwell and a dimly lit room. The first is an environmental barrier for the patient with a mobility impairment while the second might be an environmental barrier for the patient with impaired vision.

### Simulation of changing building conditions

We developed this experiment to demonstrate the algorithm's ability to correctly re-route a patient in the presence of changing building conditions. This capability could be important in an emergency situation where the number of available paths might be suddenly reduced, due, for example, to door closures. In this experiment, a simulated patient with no physical disabilities and no topographical disorientation (*P*_*C *_= 0) walked from an origin (node 1) to a destination (node 13) in Figure 2. While the patient was walking, the conditions on the shortest distance path were altered, such that the weight on an upcoming link was substantially increased, i.e. the path became inaccessible.

### Simulation of a complex scenario

Combining all the patient and building conditions mentioned above, we simulated a complex patient routing scenario. The patient had a confusion probability of *P*_*C *_= 0.6 and a mobility impairment that rendered stair climbing extremely difficult (Stair Weight = 1000). The patient was asked to navigate from node 1 to node 37. In addition, building conditions were dynamic. The weight on the link between nodes 7 and 12 escalated when the patient reached node 7, forcing the routing system to find an alternate route for the patient. In addition to the recommendations from the routing algorithm, the program would reverse the patient's direction whenever the patient simulator randomly selected a link with a very large weight (999 or greater in this simulation), denoting an inaccessible or hazardous path for the patient. In a real system, this function would be enabled via a patient localization system that would detect the approach towards the restricted area and subsequently instruct the patient to reverse directions.

## Results

The results of routing the patients with topographical disorientation are listed in Table 1. The histograms for the three measures in Table 1 were positively skewed and hence we report maximum likelihood estimates for location and spread according to a gamma distribution. We can appreciate that the patient who followed all the instructions (i.e., zero confusion probability, *P*_*C *_= 0), arrived at the desired destination with the least effort. It is interesting to note that even patients who only followed a subset of instructions (*P*_*C *_≤ 0.75), traversed significantly fewer nodes and experienced a lower travel cost than the patient who wandered randomly. In fact, the results suggest that as long as the patient follows at least one in ten instructions (*P*_*C *_< 0.9), he or she will reap some cost-savings over the random walk scenario. Generally, the more prone a patient is to random navigation, the greater the effort to reach the desired destination. We also remark that the variability of the results in Table 1 increases with rising confusion probability, *P*_*C*_.

**Table 1:** Simulation of patients with different levels of disorientation.

**Confusion probability ***P*_*C*_	**Average no. of nodes traversed**	**Average travel cost**	**Average no. of random decisions made**
1 (Random walk)	6.512 (4.149)	351.97 (237.64)	6.512 (4.149)
0.9	6.027 (3.561) *p *= 0.143	315.676 (198.72) *p *= 0.0141	5.406 (3.403)*
0.75	5.161 (2.8593)*	254.37 (147.65)*	3.858 (2.92)*
0.5	4.239 (1.9157)*	183.54 (79.79)*	2.109 (1.7476)*
0.25	3.475 (1.0366)*	144.57 (43.128)*	0.829 (0.998)*
0	3*	121*	0*

The numbers in the parentheses are the spread values according to a gamma distribution. * denotes *p *<< 10^-6^

Therefore, it appears that low values of *P*_*C *_lead to greater consistency in the selected route. Clinically, this suggests that adhering to the Dijkstra recommendations may provide the patient with a greater chance of internalizing a specific, consistent route.

Table 2 contains the simulation results for patients with disability. In the last two columns, the paths taken by each patient in each of the two scenarios is summarized by listing the nodes. It can be seen that each patient successfully avoided the target environmental barrier.

**Table 2:** Simulation of patients with different disabilities on two routes with different barriers.

**Patient**	**Weights**		**Route selected **(*source *→ *destination*)	
	
	*Mobility limitation*^1^	*Visual limitation*^1^	*1 *→ *23* *barrier: poor illumination *@ *node 9*	*8 *→ *36* *barrier: stairs between nodes 19 and 32*
*Non disabled*	0	0	1-4-5-10-9-15-23	8-7-12-19-32-38-39-36
*Mobility impairment*	1000	0	1-4-5-10-9-15-23	8-7-12-19-20-21-22-41-40-39-36
*Visual impairment*	0	1000	1-4-5-6-7-12-19-18-17-16-15-23	8-7-12-19-32-38-39-36

^1 ^The mobility and visual limitations were captured by the "Stair Difficulty" and "Poor Illumination" fields, respectively, in the patient table.

**Figure 4:**
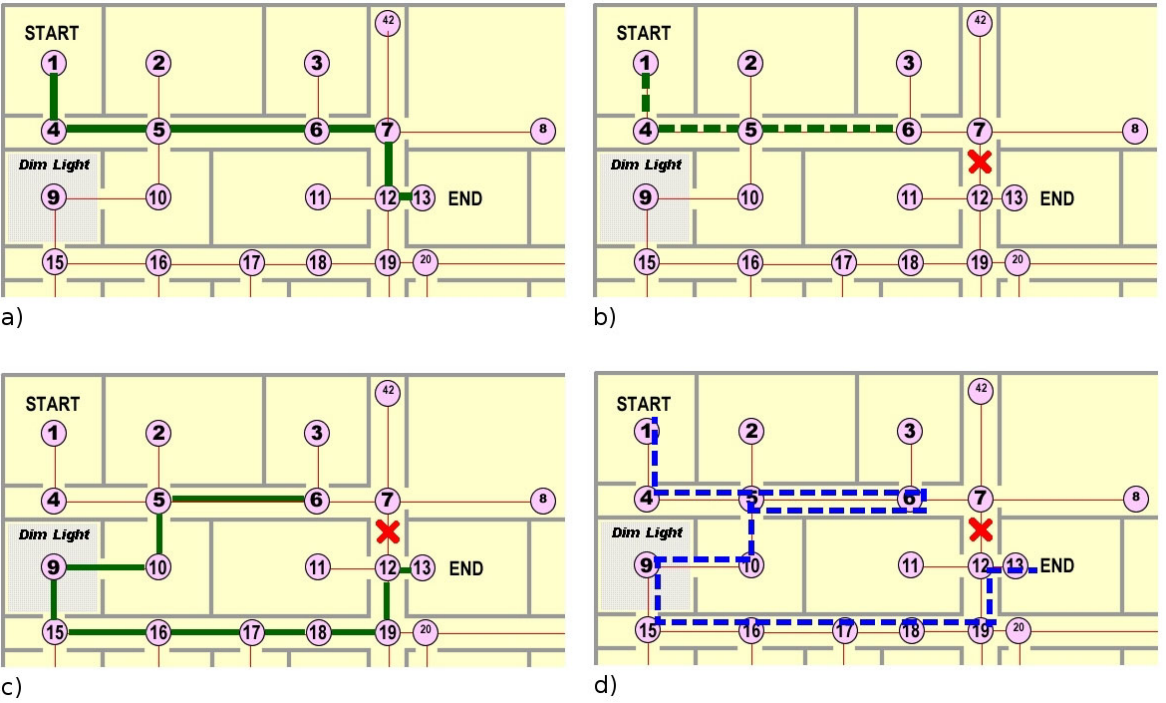
Routing results with changing building conditions. A solid line depicts the recommended route while a dashed line highlights the actual path traversed. (a) The patient is asked to walk from node 1 to node 13. The algorithm selected the optimal route as indicated. (b) When the patient reached node 6, the edge weight between nodes 7 and 12 increased to 20 times its original value. (c) At node 6 the algorithm calculated the best alternative route. (d) The final route followed by the patient.

The simulation results for routing amid building changes is graphically represented in the four panels of Figure 4. Panel (a) shows the originally proposed route from the source (node 1) to the destination (node 13). This recommendation persisted until the patient reached node 6, at which point the link between nodes 7 and 12 became no longer available as seen in panel (b). Subsequently, the patient was re-routed from his current location (node 6) through the lengthy detour indicated in panel (c). The final route shown in panel (d) indicates that the patient was actually asked to partially retrace his steps in light of the building change.

**Figure 5:**
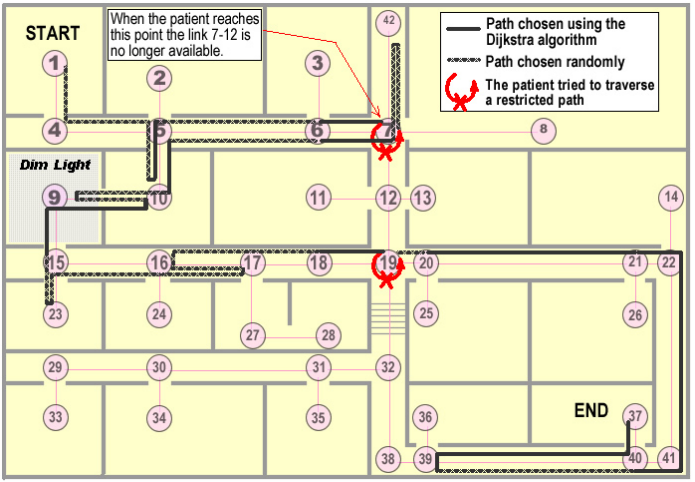
An example of the complex scenario simulation: a typical route followed by a patient with topographical disorientation and mobility impairment amid fluctuating building conditions.

A typical route-following example from the complex scenario simulation is shown in Figure 5. In this simulation, the link 7–12 becomes inaccessible part way through the patient's journey. The thick solid lines indicate decisions that the patient made in accordance with the routing algorithm instructions. The hashed lines indicate random navigational decisions, some of which coincided with the recommendations of the routing algorithm. We notice that the patient retraced his path in a few locations due to his random wandering. At node 7, the patient decided to go towards node 12, a link which was no longer available. Later on, at node 19, the patient attempted to access the stairway, which was an identified environmental barrier. In both of these instances, the patient was provided with the instruction to reverse his direction. Clearly, the patient did not follow to a tee his recommended shortest path i.e., 1-4-5-6-7-6-5-10-9-15-16-17-18-19-20-21-22-41-40-37 with a total cost of 1843. Nonetheless, he still reached the desired destination carving out a route that oscillated about the optimal path, racking up a final cost of 3157, which is still 38% less than the average cost of a random walk in this context.

## Discussion

From the disorientation simulation, we see that even a patient who follows a subset of navigational directions, will benefit in terms of reduced distance and time of travel. The examples also illustrate that the proposed routing scheme can adapt to different patient abilities, environmental barriers and dynamic modifications of the indoor pathways.

### Potential clinical applications

The proposed algorithm could be deployed in a patient navigation system where weights of certain links in the connected graph are automatically updated at various times in the day. Target populations would include patients with topographical disorientation, patients with different physical abilities and healthcare staff, families or visitors needing to navigate an unfamiliar indoor environment. Alternatively, the routing algorithm might be connected to an alarm system and provide a set of weights according to the nature of the emergency. For example, a link traversing elevators might have a very high weight whenever a fire alarm is triggered. A patient would then be routed away from the elevator unless there were no other navigational options for the particular patient. This might be the case for a patient who uses a wheelchair, in which case, the physical barriers of stairs would retain a higher weight than elevators even in the event of a fire.

The algorithm always recalculates the optimal route between the current and destination nodes. Therefore, assuming navigational instructions are followed, it would be known *a priori *whether or not the patient would have to traverse a link with a high weight value. The routing system, if connected to a network, could generate a message to request assistance at the forthcoming link. In this way, appropriate health care personnel could be dispatched to provide the required assistance at the specified location.

### Limitations and future work

The algorithm has only been demonstrated via computer simulation with simplified patients and a subset of environmental challenges. Clinical tests with human participants are necessary to comprehensively characterize patient behaviors and potential barriers, including, for example, auditory and visual distractions, nonstationary landmarks, and crowded spaces. Also, unaccounted for at present are patient preferences, which may serve to break ties between two competing routes of otherwise equal cost. Fortunately, the proposed system is scalable in the sense that the database could easily embody additional details about the patient and the environment.

In the above examples, the graphs were generated by manual placement of nodes. In sophisticated or large scale floor plans, it may be advantageous to develop automatic graph generation methods as in the geoinformatics literature (e.g., [29]). Further, we have only described routing on a one level building. Generalizing the method for multilayer routing, for example, using 3-dimensional graphs, would be useful in hospital environments where patients are permitted limited interlevel travel.

The assignment of weights to characterize physical barriers and the patient's ability to overcome these barriers has been arbitrary in our simulations. More realistic weight determination needs to be established, as suggested through standardized assessments, building measurements and architectural drawings. Further, future work should explore a means for physicians or occupational therapists to set patient-specific weight values in an intuitive way, for example, via a series of sliders whose positions indicate the patient's ability to negotiate specific barriers.

In the current implementation, the patient can receive new directions only upon arrival at a new node, leaving "dead spaces" between nodes where the algorithm offers no new information. The amount of tolerable dead space would be patient and building dependent and would likely necessitate therapist assessments. Algorithmically, the spatial granularity of the information service can be easily refined by adding more intermediate nodes.

## Conclusion

We have presented a method of routing patients with topographical disorientation through an indoor environment, accounting for physical abilities of the patient, environmental barriers and dynamic building changes. The routing algorithm and database could be integrated into wearable and mobile platforms within the context of an ambient intelligence solution.

## Competing interests

The author(s) declare that they have no competing interests.

## Authors' contributions

JT designed the routing algorithm, the software tools and data structures for the experiments. JT proposed the initial design of experiments, executed them, and analyzed and interpreted the data. JT worked on the initial draft of the manuscript. TC advised upon the design and coordination of the study, experiments and data analysis, and multiple revisions of the manuscript. Both authors read and approved the final version of the manuscript.
